# An updated systematic review and meta-analysis of tooth loss in patients with periodontitis and the risk of mild cognitive impairment

**DOI:** 10.3389/froh.2026.1710871

**Published:** 2026-03-18

**Authors:** Congcong Zou, Haibei Liu, Lingli Li, Zhenzhen Lin, Yanhua Qiu

**Affiliations:** 1 Department of Anesthesiology, West China Hospital, Sichuan University, Chengdu, China; 2 Anesthesia and Surgery Center of West China Xiamen Hospital, Sichuan University, Xiamen, China

**Keywords:** meta-analysis, mild cognitive impairment, periodontal diseases, periodontitis, tooth loss

## Abstract

**Objective:**

Conflicting evidence has shown a connection between tooth loss, periodontitis, and increased risk of mild cognitive impairment (MCI); thus, we conducted a meta-analysis to clarify this association.

**Materials and methods:**

PubMed, Embase, Web of Science, the Cochrane Library, and MEDLINE (up to March 2025) were searched. Cross-sectional and longitudinal studies were included. Fully-adjusted odds ratios (aORs) with the most severe form of periodontitis or tooth loss were pooled with a random effects model using STATA 18/MP software. Subgroup and sensitivity analyses were conducted to elucidate possible heterogeneity.

**Results:**

The meta-analysis of five studies showed that neither periodontitis nor tooth loss substantially increased MCI risk after full adjustment, with pooled aORs of 1.27 (95% CI: 0.87–1.86, *I*^2^ = 63.9%) and 1.22 (95% CI: 0.97–1.54, *I*^2^ = 53.8%), respectively. A significant association was found between periodontitis and MCI with low heterogeneity in the Centers for Disease Control and Prevention-American Academy of Periodontology diagnostic criteria for periodontitis subgroup (aOR of 2.78, 95%CI 1.54–5.02, *I*^2^ = 0.0%) and between MCI and significant tooth loss (aOR of 1.50, 95%CI 1.15–5.95, *I*^2^ = 0.0%). The sensitivity result confirmed the robustness of the results.

**Conclusion:**

Overall, periodontitis and tooth loss were not statistically associated with increased MCI risk. Whereas the association between severe periodontitis and significant tooth loss and MCI was statistically significant. Thus, severe periodontitis and significant tooth loss were linked to decreased cognitive function.

**Systematic Review Registration:**

PROSPERO CRD420251055491.

## Introduction

Mild cognitive impairment (MCI) is a nuanced gray area between intact cognitive functioning and clinical dementia (1) or Alzheimer’s disease (AD) (2, 3). Defined as a marker of the initial phase of progressive memory decline, MCI does not interfere with one’s ability to perform activities of daily living and does not meet the diagnostic criteria for dementia or AD, and is widely regarded as an intermediate state of cognitive function between normal aging and dementia or AD (2–4). An overall MCI prevalence of 12%–22% has been reported among 60-year-old community-dwelling adults, and a prevalence of 16% of MCI has been found in residents over 70 years old (3, 5–9). The progression rate from MCI to any form of dementia was reported to be 3–5 times higher than in the general population with normal cognition (10). As a transitional stage of early cognitive impairment, MCI has drawn significant attention to its various risk factors, such as (1) the non-modifiable risk factors of aging and genetic risk factors (11–14); (2) lifestyle factors such as smoking and central obesity (15), unhealthy dietary habits such as high saturated fat and low vegetable consumption, low education levels (16, 17), and social and mental inactivity (18–21); and (3) comorbid cardiovascular diseases such as diabetes mellitus (22), coronary heart disease (23), hyperlipidemia (16), and hypertension (10). Moreover, reports have demonstrated the association between tooth loss, periodontitis, and the onset of MCI, dementia, or AD (4, 24–30).

Oral diseases are the most prevalent non-communicable diseases, affecting a staggering 3.9 billion people worldwide (31, 32). Untreated dental caries, severe periodontitis, and edentulism have been the three most common and impactful oral conditions, with their prevalences increased by 53%, 91.5%, and 100% between 1990 and 2021, respectively (31). They impair an individual's normal physical and social functioning, including chewing, taste, speech, appearance, and facial expressions, and exacerbate economic burdens. While oral diseases impact individuals throughout their entire lifespan, older populations are particularly prone to tooth loss and edentulism (31). As the WHO projects that the proportion of people aged 60 and over will rise from 12% in 2015 to 22% in 2050, tooth loss is the leading cause of disability-adjusted life years (DALYs) (33). Periodontitis, one of the main causes of tooth loss, is a ubiquitous, chronic, and irreversible inflammatory condition (34). It is linked to many factors, including chronic conditions such as stroke and diabetes, as well as medications. Minor changes to the heavy burden in the last three decades indicate inefficient efforts to control oral diseases, including dental caries, tooth loss, and periodontitis.

Several studies have explored whether tooth loss or periodontal health was correlated with cognitive dysfunction or dementia or AD, with conflicting conclusions due to differences in inclusion criteria (26, 35–37), study design (4, 37–39), statistical analysis (27), and diagnostic criteria (4, 39, 40). Additionally, increasing evidence has suggested a double-direction relationship between poor oral status and the onset of dementia in elders (36, 41–45). However, there is a scarcity of published research investigating the association between both tooth loss and periodontal disease and the risk of MCI. This systematic review aimed to consolidate and strengthen evidence regarding the increased risk of MCI in the elderly population resulting from tooth loss combined with periodontal disease.

## Materials and methods

According to the principles of Preferred Reporting Items for Systematic Reviews and Meta-analysis (PRISMA) (46), we conducted this systematic review with rigorous methodology in order to minimize the risk of bias. The research has been registered at the international Prospective Register of Systematic Reviews (PROSPERO). (CRD420251055491, available from https://www.crd.york.ac.uk/PROSPERO/view/CRD420251055491).

### Search strategies

The PEO principle was used to focus the research question. The population (P) was adults without signs of cognitive decline, Alzheimer's disease, or dementia of any type at baseline; the exposure (E) was periodontitis and tooth loss diagnosed by well-trained dentists through professional clinical examination; and the outcome (O) was MCI/cognitive decline/mild memory impairment (MMI) evaluated through verified tests. More details were described in the registered protocol as mentioned above. We searched PubMed, Embase, Web of Science, MEDLINE, and the Cochrane Library for abstracts and full-text papers published from inception to 25 March 25 2025. Both MeSH terms (“tooth loss,” “periodontitis,” “chronic periodontitis,” “cognitive dysfunction”) and entry terms (e.g., “missing tooth,” “edentulism dentition,” “loss of teeth,” “periodontal disease,” “cognitive decline,” “cognitive deterioration,” “mild memory loss”) were used in the search strategies. More detailed information is presented in Supplementary Appendix 1. We also screened additional records from references and websites, but found no records to include.

### Inclusion criteria

The inclusion criteria were as follows: longitudinal prospective cohort and cross-sectional studies published up to and including 25 March 2025; the exposure factors were descriptions of chronic periodontal disease and tooth loss, with at least one of these having been professionally assessed and recorded by a standardized process during a clinical examination; and the included individuals had been were diagnosed with MCI through verified tests such as the Mini-Mental State Examination (MMSE) and the digit symbol substitution test (DSST). Finally, effect sizes, such as adjusted odds ratio (aOR), adjusted β (Aβ), and adjusted hazard ratio (aHR), that described the association between tooth loss, periodontitis, and MCI were available for extraction.

### Exclusion criteria

We also set the exclusion criteria as follows: (1) studies that assessed the influence of dementia or AD on tooth loss or on periodontitis and (2) studies with outcomes other than MCI or with exposure factors other than periodontitis or tooth loss (such as smoking, diabetes, and other risk factors common to both diseases). These factors can be regarded as potential hidden confounders, thus constituting a limitation of this review. Additionally, studies that collected exposure information through non-clinical examinations, such as surveys or interviews, were excluded, as were case-control studies, case reports, reviews, and animal studies.

### Selection process

**Figure 1:**
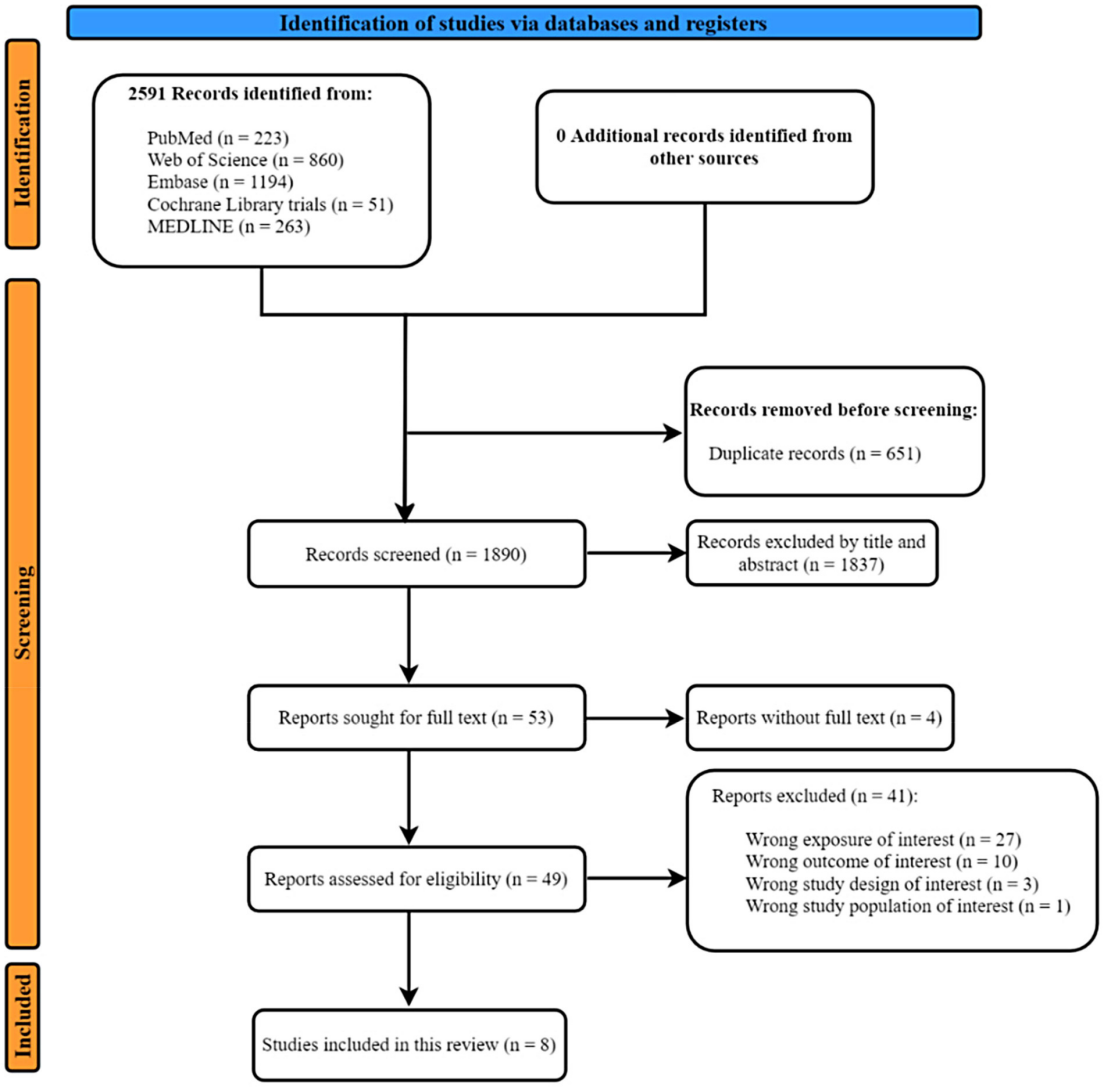
Flowchart of the included studies.

Following the initial search, all the retrieved results were imported into reference management software, namely Covidence (https://www.covidence.org/), and duplicate records were automatically screened out. The remaining records with titles and abstracts were then screened by two independent reviewers (CZ and HL). A third reviewer resolved any disagreements (YQ) during the process. We calculated the interagreement score at each stage (Cohen's *κ* score) (47). Figure 1 illustrates the main selection process.

### Data extraction process

According to the Cochrane Handbook (48), the two independent reviewers collected data using a customized data extraction form. For the purpose of this review, the following information was extracted and tabulated for all the selected studies (Table 1): title; first author's name; publication year; study design; population description; study location; recruiting method; study objective; start and end date of data collection; total duration of observation; sample size at baseline; withdrawals and dropouts during follow-up time; age at baseline recruitment; gender; methods used to evaluate periodontal health, such as measurements of probing depth and alveolar bone height in radiographs; the measures used to assess the extent of severity of disease; the number of remaining teeth measured at baseline and at follow-up; the number of missing teeth (calculated as the difference between the number of remaining teeth measured at baseline and at follow-up); cognitive function assessment tests; and study conclusions.

**Table 1:** Characteristics of the included studies.

Study authors	Study design	Location of study	Population description	Number of participants	Age at baseline	Sex	Withdrawals and dropouts	Data collection in	Start and end date	Method for assessing periodontal health	Method for assessing tooth loss	Mild cognitive impairment assessment		Conclusion
Kaye et al. 2010 (52)	Prospective cohort study	USA	Male U.S. veterans	597	24–84	Males	634 (51.5%)	8 years	1993–2001	Number of teeth showing an increase in PPD by ≥2 mm from baseline; number of teeth showing a decrease in bone height by ≥40% from baseline	Number of teeth lost per decade of follow-up	MMSE (MCI<25)	SCT (MCI<10)	Risk of cognitive decline in older men increases as more teeth are lost. Periodontal disease and caries (major reasons for tooth loss) are also related to cognitive decline.
Okamoto et al. 2015 (51)	Prospective cohort study	Japan	Elderly residents of Nara	2,335	≥ 65	Males and females	1,361 (36.8%)	5 years	2007–2012	CPI on 10 representative teeth in the 6 segments of the oral cavity codes 0–4. The highest code level was regarded as the maximum CPI code.	Number of remaining teeth divided into five groups at baseline and follow-up: 1–8, 9–16, 17–24, and 25–32.	MMI status: MMSE ≥24 plus	Word recall score ≤1 plus GDS score ≤5	Tooth loss predicts the development of MMI among the elderly.
Nilsson et al. 2017 (57)	Cross-sectional study	Sweden	Older adults (SNAC cohort)	775	60–99	Males and females	NA	Ongoing since 2001	2001–2003	Proportion of teeth with ≥5 mm PPD on>30% of teeth and ALB (distance from CEJ to alveolar bone)>4 mm on OPG at ≥30% of sites	Number of remaining teeth divided into two groups: 1–19/≥20	MMSE (MCI<25)	Clock test (MCI<8)	A history of periodontitis and tooth loss may be of importance for cognitive function among older adults.
Luo et al. 2023 (53)	Prospective cohort study	USA	Hispanic/Latino immigrants	5,709	50–74	Males and females	668 (10.5%)	18 years	2006–2024	No/mild/moderate/severe disease based on interproximal attachment loss (PPD) and ABL according to CDC-AAP definition	STL counted as eight or more teeth lost at follow-up compared to baseline	Various neuropsychiatric battery tests including six item screener, verbal learning tests, word fluency, digit symbol tests, and trail making tests		Significant tooth loss is a significant risk factor for mild cognitive impairment.
Gu et al. 2023 (54)	Cross-sectional study	China	Chinese rural older adults	677	55–65	Males and females	227 (25.1%)	Ongoing since 2013	2013–2020	No/mild/moderate/severe disease based on interproximal attachment loss (PPD) and ABL according to a half-reduced CDC-AAP definition	Number of missing teeth	The Chinese version of MMSE (MCI<25)	The Beijing version of MoCA (MCI<26)	Poor periodontal status was strongly associated with worse global cognition performance, especially in the short-term memory and executive domains in the aging population.
Yang et al. 2023 (56)	Cross-sectional study	China	Older adults in aged care facilities	371	≥65	Male and female	NA	Ongoing since 2020	2020–2021	BGI based on periodontal probing depth and bleeding on probing	Number of remaining natural teeth	MMSE (MCI<26.5 for those aged ≤75 years and education ≤6 years; MCI<22.5 for those aged>75 years and education ≤6 years; MCI<28.5 for those aged ≤75 years and education>6 years; MCI<26.5 for those aged>75 years and education>6 years)		Periodontitis and multiple tooth loss were factors associated with cognitive impairment.
Gao et al. 2023 (49)	Cross-sectional study	USA	NHANES participants	1,987	≥60	Males and females	521 (20.7%)	NA	2011–2012, 2013–2014	No/mild/moderate/severe disease based on interproximal attachment loss (PPD) and ABL according to CDC-AAP definition	Number of missing teeth	The global cognitive function is based on the sum of the CERAD W-L score, DSST score, and GCF score.		Robust bidirectional associations between oral disease and cognitive function among the aging population.
Ye et al. 2024 (55)	Cross-sectional study	China	older adults from Wuhan hospitals	234	≥65	Male and female	NA	NA	2023	Yes/no according to CDC-AAP definition	Number of teeth lost	MMSE (MCI<17 for illiterate; MCI<20 for primary school, MCI<20; MCI<24 for junior high school and above)	MoCA (MCI < 26)	OHRQoL was found to be associated with MCI.

U.S., United States; SNAC, Swedish National Study on Aging and Care; DLS, dental longitudinal study; PPD, probing pocket depth; ABL, alveolar bone loss; MMSE, the mini-mental state examination; SCT, a spatial copying task; MCI, mild cognitive impairment; CPI, community periodontal index; WHO, World Health Organization; GDS, geriatric depression scale short version; CEJ, cement-enamel junction; OPG, orthopantomography; CDC, Centers for Disease Control and Prevention; AAP, American Academy of Periodontology; STL, significant tooth loss; BGI, the Biofilm-Gingival Interface index; CERAD W-L, Consortium to Establish a Registry for Alzheimer's Disease World Learning subtest; DSST, digit symbol substitution test; GCF, Global Cognitive Function; OHRQoL, oral health-related quality of life.

### Assessment of quality

The quality of all non-randomized studies was assessed using the Newcastle–Ottawa Quality Assessment Scale, which comprises eight items across three domains in accordance with Agency for Healthcare Research and Quality (AHRQ) standards (48). The quality ratings were defined as follows: Good/high quality: three or four stars in the selection domain, one or two stars in the comparability domain, and two or three stars in the outcome/exposure domain; fair/moderate quality: two stars in the selection domain, one or two stars in the comparability domain, and two or three stars in the outcome/exposure domain; and low/poor quality: zero or one star in the selection domain, zero stars in the comparability domain, or 0 or one stars in the outcome/exposure domain.

### Quantitative analysis

In addition to a descriptive synthesis, we conducted a meta-analysis using STATA version 18.0./MP (STATA Corp, College Station, TX, USA). Although eight studies were included in the analysis, only five were available for the meta-analysis. In the observational studies, there were both unadjusted and adjusted ORs at different levels. Due to differences in the type and number of confounding factors, we used multiple models for the extracted adjusted OR values. In this meta-analysis, we conducted statistical pooling and discussion of the unadjusted OR values and OR values with the most confounding factors, respectively. When different severe forms of tooth loss and periodontitis were reported in individual studies, the effect sizes (aHRs and aORs) corresponding to the highest disease severity scores were extracted to convert into log(OR) values for analysis. The *β* linear regression coefficients extracted from Gao et al. (49) were not amenable to direct conversion into ORs as the two measures rely on disparate model assumptions and are tailored to different types of dependent variables. Considering the high clinical heterogeneity among the observational studies, a random effects model was applied. Heterogeneity was measured using the chi-squared test (*I*^2^) with a level of significance of *p* < 0.05.

### Additional analysis

We also analyzed the sources of heterogeneity using subgroup and sensitivity analyses. According to differences among different diagnostic criteria and cutoff values for periodontitis, the studies were divided into a Centers for Disease Control and Prevention-American Academy of Periodontology (CDC-AAP) diagnostic criteria group and other diagnostic criteria group for analysis. Tooth loss was also categorized into two groups, namely, the loss of one tooth and the significant tooth loss groups, according to the different levels of significant tooth loss that were described in each study. Moreover, study design has an impact on heterogeneity; thus, the studies were divided into a cross-sectional study group and a cohort study group. The stability of the adverse effect of periodontitis or tooth loss on MCI was also verified using the leave-one-out method as a sensitivity analysis. Since the total number of included studies was less than 20, both Egger's and Begg's tests for publication bias would have limited test power and sensitivity; thus, we did not create funnel plots and conduct related tests (50).

## Results

A total of 2,591 records were found initially, as shown in Figure 1. Subsequently, 651 duplicate records were automatically screened out and 1,837 of the remaining records with titles and abstracts were excluded by two independent reviewers. During the second round of screening, 53 reports remained, as four were excluded due to the unavailability of full texts. Of the remaining articles, 41 were rejected (the specific reasons are detailed in Supplementary Appendix 2). Finally, eight studies were included. The kappa score (*K* score = 0.91) showed substantial interagreement between the reviewers throughout the whole screening process.

### General characteristics

Table 1 presents a summary of the characteristics of the eight selected studies. In terms of study design, three of the eight studies were prospective cohort studies (51–53) and five were cross-sectional studies (49, 54–57). A total of 12,685 participants were recruited at baseline across four countries (China, Sweden, the USA, and Japan). The participants’ age in the majority of the studies was over 50 years at baseline, with one study (52) including younger males aged 24 years and older. Periodontitis and tooth loss were identified as the primary and secondary exposures, and all the studies assessed oral health status through clinical examinations conducted by at least two independent, qualified, and calibrated professionals, such as dentists, hygienists, and periodontists. The studies evaluated tooth loss over the years using different approaches. The majority of the studies examined the severity and progression of periodontal disease, while half of the included studies (49, 53–55) assessed the severity of periodontitis as no/mild/moderate/severe according to the CDC-AAP criteria (58). The definition of cognitive outcomes also varied across the studies. Nevertheless, the definition used to describe mild memory impairment (MMI) was comparable to that of MCI (51). The diagnosis of cognitive decline was predominantly assessed using the MMSE, with an MMSE score of 21–25 indicating MCI, which was the outcome of interest.

Table 2 shows that periodontal disease progression was associated with an increased risk of mild cognitive deterioration. Overall, 8%–71% of participants who had normal MMSE scores at baseline fell below the cutoff point upon repeat testing (mainly MMSE <25), secondary to tooth loss and poor periodontal status (51, 52, 54–57). Table 3 shows a strong association between tooth loss and a heightened risk of MCI.

**Table 2:** Risk of cognitive impairment in relation to periodontal disease's progression.

Study	Exposure	Exposure cutoff point	Adjustment	Study estimate	95%LL	95%UL	*P*
Kaye et al. 2010 (52)	Each additional tooth loss with ABL progression/decade	40% increase from baseline or teeth lost	History of CHD, average alcohol intake, number of teeth with <60% bone loss at baseline	1.03 (HR)	1.00	1.07	NA
	Each additional tooth loss with PPD progression/decade	≥2 mm from baseline or teeth lost	History of CHD, average alcohol intake, number of teeth with <3-mm pocket depth at baseline	1.04 (HR)	1.01	1.09	NA
	Each additional tooth loss with new caries or restorations/decade	Developed caries after baseline, restored or lost	History of CHD, average alcohol intake, number of sound teeth at baseline	1.02 (HR)	0.97	1.08	NA
Okamoto et al. 2015 (51)	CPI codes 0–4	Code 4	Age, gender, MMSE-total, recall, GDS, education length, alcohol intake, smoking habits, history of cancer, myocardial infarction, cerebrovascular disease, diabetes mellitus, hypertension, dyslipidemia, and follow-up systolic and diastolic blood pressure and cerebrovascular disease	1.04 (AOR)	0.74	1.47	0.828
Nilsson et al. 2017 (57)	Distance from CEJ to marginal bone level	≥ 4 mm on ≥30% sites	Age, gender, education	2.7 (AOR)	1.2	5.9	0.013
Luo et al. 2023 (53)	% of teeth with attachment loss and % of teeth with increased PPD	Mild (≥2 interproximal sites with AL ≥3 mm and ≥2 sites with PD ≥4 mm (not on the same tooth, or ≥1 site with PD ≥5 mm.)	Age, gender, race, education, income, health insurance, time since visit 1, field centers, body mass index, smoking status, alcohol use, dietary quality, depression, hypertension, stroke, myocardial infarction, diabetes, hs-CRP	1.41 (AOR)	0.84	2.36	0.20
		Moderate (≥2 interproximal sites with AL ≥4 mm[not on the same tooth], or ≥2 interproximal sites with PD ≥5 mm [not on same tooth)		0.76 (AOR)	0.56	1.04	0.08
		severe [≥2 interproximal sites with AL ≥6 mm (not on same tooth), and ≥1 intreproximal sites with PD ≥5 mm]		1.01 (AOR)	0.66	1.55	0.95
Gu et al. 2023 (54)	% of teeth with attachment loss, and % of teeth with increased PPD	No PeD, mild PeD:≥1 interproximal sites with CAL ≥3 mm, along with ≥1 interproximal sites PD ≥4 mm; moderate PeD: ≥1 interproximal sites CAL ≥4 mm, or ≥1 interproximal sites PD ≥5 mm; severe PeD:≥1 interproximal sites (not the same tooth) CAL ≥6 mm, along with ≥1 interproximal sites PD ≥5 mm; Complete edentulism.	Age, gender, education year, toothbrushing frequency, smoking alcohol consumption, hypertension, diabetes, hyperlipidemia	0.87 (AOR)	0.58	1.30	NA
Yang et al. 2023 (56)	the Biofilm-Gingival Interface (BGI) index	BGI >2	Age, gender, education	2.89 (AOR)	1.2	6.95	0.018
Gao et al. 2023 (49)	% of teeth with attachment loss, and % of teeth with increased PPD	No PeD, mild PeD: ≥1 interproximal sites with CAL ≥3 mm, along with ≥1 interproximal sites PD ≥4 mm; moderate PeD: ≥1 interproximal sites CAL ≥4 mm, or ≥1 interproximal sites PD ≥5 mm; severe PeD: ≥1 interproximal sites (not the same tooth) CAL ≥6 mm, along with ≥1 interproximal sites PD ≥5 mm; Complete edentulism.	Age, gender, education qualification, ethnicity, marital status, poverty index ratio, anthropometric measures, smoking status, alcohol intake, substance misuse, physical activity, intake of sugar, carbohydrate, energy, cardiovascular diseases, diabetes, liver disease, arthritis, depression, sleep disorder, dental hygiene behavior	−0.39 (Aβ)	−0.69	−0.1	NA

CHD, coronary heart disease; PPD, probing pocket depth; ABL, alveolar bone loss; CPI, community periodontal index; GDS, geriatric depression scale short version; CEJ, cement-enamel junction; BGI, Biofilm-Gingival Interface index.

**Table 3:** Risk of cognitive impairment in relation to tooth loss.

Study	Exposure	Exposure cutoff point	Adjustment	Study estimate	95%LL	95%UL	*P*
Kaye et al. 2010 (52)	Number of teeth lost per decade of follow-up	Each additional tooth lost/decade	History of CHD, average alcohol intake, number of teeth with <60% bone loss at baseline	1.09 (HR)	1.01	1.18	NA
Okamoto et al. 2015 (51)	Number of remaining teeth	Each tooth lost at follow-up	Age, gender, MMSE-total, recall, GDS, education length, alcohol intake, smoking habits, history of cancer, myocardial infarction, cerebrovascular disease, diabetes mellitus, hypertension, dyslipidemia, and follow-up systolic and diastolic blood pressure, and cerebrovascular disease	1.04 (AOR)	0.98	1.11	0.201
Nilsson et al. 2017 (57)	Number of remaining teeth	1–19 teeth remaining	Age, gender, education	2.0 (AOR)	1.1	3.6	0.03
Luo et al. 2023 (53)	Significant tooth loss	≥ 8 teeth lost	Age, gender, race, education, income, health insurance, time since visit 1, field centers, body mass index, smoking status, alcohol use, dietary quality, depression, hypertension, stroke, myocardial infarction, diabetes, hs-CRP	1.36 (AOR)	1.01	1.85	0.04
Gu et al. 2023 (54)	Number of missing teeth	Each tooth lost	Age, gender, education year, toothbrushing frequency, smoking alcohol consumption, hypertension, diabetes, hyperlipidemia	1.05 (AOR)	0.70	1.57	NA
Yang et al. 2023 (56)	Multiple tooth loss	Number of natural teeth ≤9 remaining	Age, gender, education	2.62 (AOR)	0.55	12.48	0.225
Gao et al. 2023 (49)	Number of missing teeth	Each tooth lost	Age, gender, education qualification, ethnicity, marital status, poverty index ratio, anthropometric measures, smoking status, alcohol intake, substance misuse, physical activity, intake of sugar, carbohydrate, energy, cardiovascular diseases, diabetes, liver disease, arthritis, depression, sleep disorder, dental hygiene behavior	−0.04 (Aβ)	−0.06	−0.02	NA

### Quality evaluation

Based on the Newcastle–Ottawa scale scoring, among the eight studies, one was deemed to be of low quality (51), two were classified as fair or moderate quality (56, 57), and five were rated as good/high quality (49, 52–55) (Supplementary Appendix 3).

### Association between periodontitis and MCI

**Figure 2:**
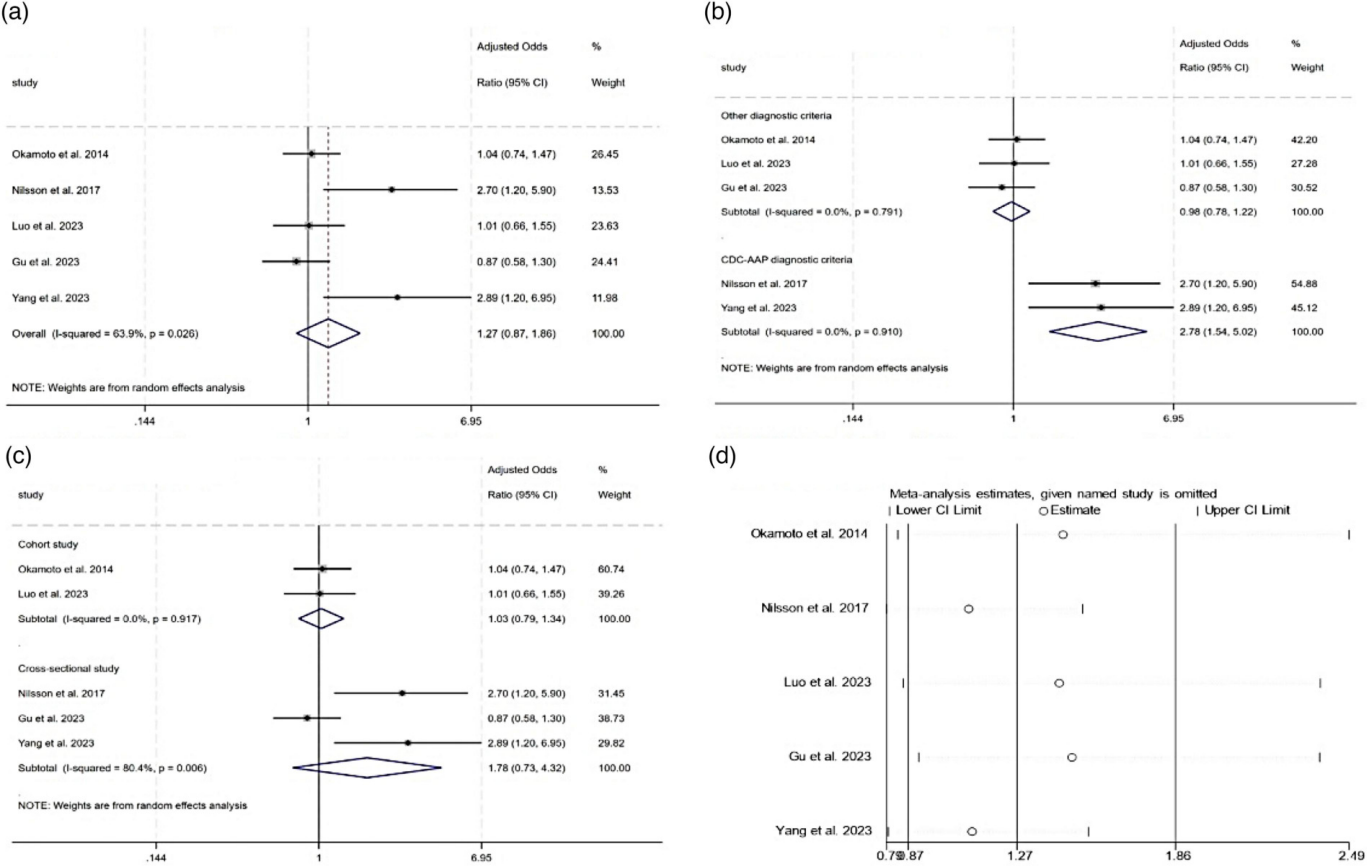
Meta-analysis of the association between periodontitis and MCI. **(a)** Forest plot of the association between periodontitis and MCI. **(b)** Subgroup analysis of the association between periodontitis and MCI. **(c)** Subgroup analysis of the association between periodontitis and MCI. **(d)** Sensitivity analysis of the effect of periodontitis on MCI.

The pooled aORs from five studies indicated that periodontitis was associated with an elevated risk of developing MCI, though this association lacked statistical significance (Figure 2a) (51, 53, 54, 56, 57). The most severe form of periodontitis was not associated with a significantly elevated risk of cognitive decline (pooled aOR = 1.27, 95% CI 0.87–1.86, *P* = 0.213), with *I*^2^ = 63.9%. Conversely, when unadjusted ORs were pooled together from five different studies (49, 53, 55–57) (Supplementary Figure S2-S1), a significant association between periodontitis and MCI was found (pooled unadjusted OR = 2.06, 95% CI 1.31–3.24, *I*^2^ = 81.6%).

A subgroup analysis indicated that the heterogeneity was predominantly due to the different diagnostic methods for periodontitis. As shown in Figure 2b, both subgroups had an *I*^2^ value of 0%. However, a subtotal aOR of 2.78 (95% CI 1.54–5.02, *P* = 0.001) was found in the CDC-AAP diagnostic criteria group, while a subtotal aOR of 0.98 (95% CI 0.78–1.22, *P* = 0.838) was found in the other group. In order to clarify the influence of study design on the pooled aOR, we performed another subgroup analysis. As shown in Figure 2c, in the cohort study group, the subtotal aOR was 1.03 (95% CI 0.79–1.34, *P* = 0.839, *I*^2^ = 0.0%), while in the cross-sectional study group, the subtotal aOR was 1.78 (95% CI 0.73–4.32, *P* = 0.205, *I*^2^ = 80.4%). A subgroup analysis of the pooled unadjusted ORs found similar results, as shown in Supplementary Figures S2-3, S2-4. Using the leave-one-out method, the sensitivity analysis verified the stability of the adverse effect of periodontitis on MCI, as shown in Figure 2d.

### Association between tooth loss and MCI

**Figure 3:**
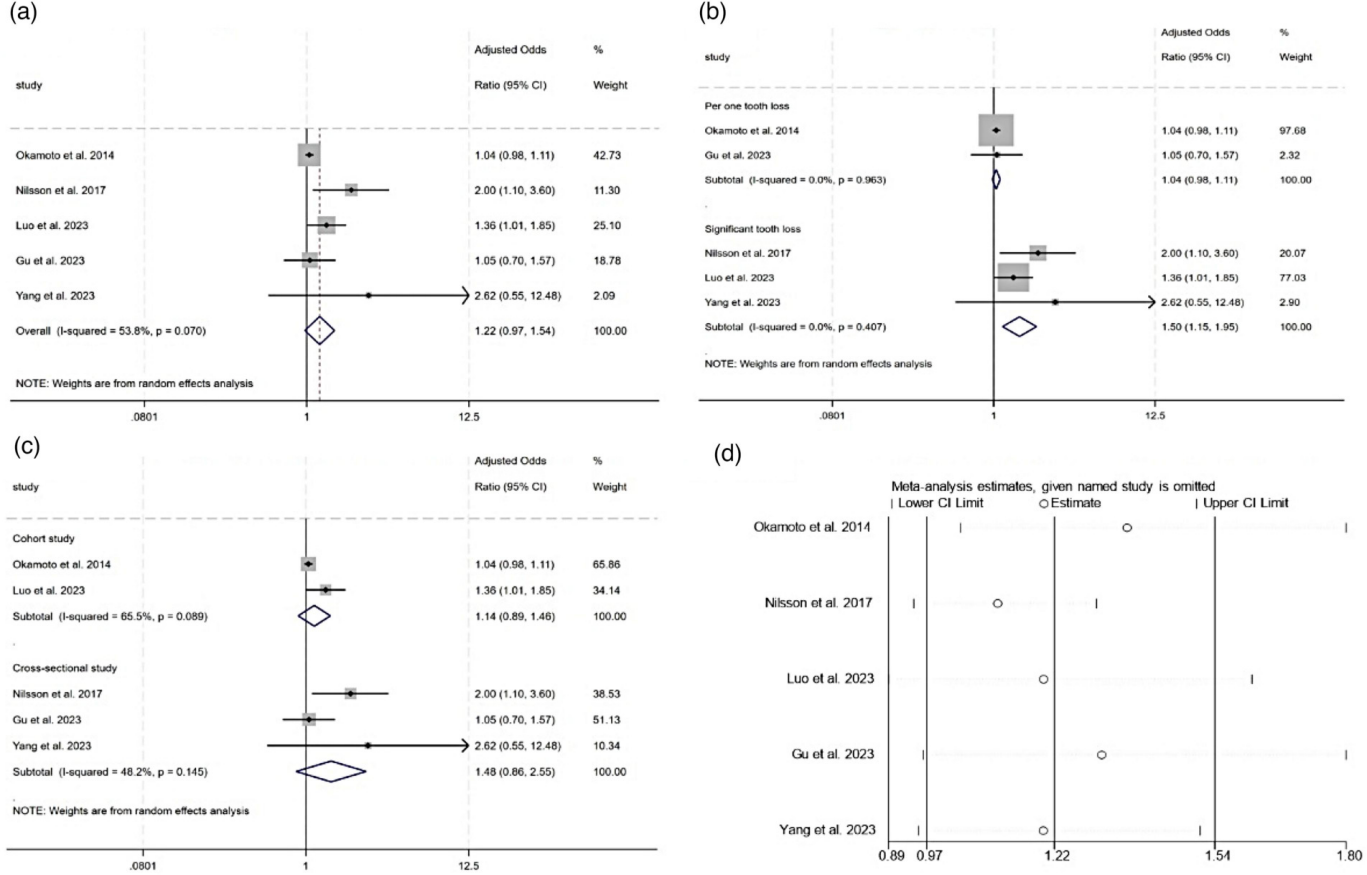
Meta-analysis of the association between tooth loss and MCI. **(a)** Forest plot of the association between tooth loss and MCI. **(b)** Subgroup analysis of the association between tooth loss and MCI. **(c)** Subgroup analysis of the association between tooth loss and MCI. **(d)** Sensitivity analysis of the effect of tooth loss on MCI.

Figure 3a illustrates that the tooth loss was not independently associated with an elevated risk of MCI, with a pooled odds ratio of 1.22 (95% CI of 0.97–1.54, *P* = 0.087) and an *I*^2^ value of 53.8% across five studies (51, 53, 54, 56, 57). Supplementary Figure S3-1 shows a pooled unadjusted OR of 1.91 (95% CI 1.31–2.78), with high heterogeneity (*I*^2^=74.7%).

In a subgroup analysis (Figure 3b), the studies were divided into two groups based on their tooth loss assessment method. Group one used the loss of one tooth during follow-up as an exposure factor (51, 54), while group two used the significant loss of at least five teeth (53, 56, 57). The results indicate that the high heterogeneity came from different methods of assessing tooth loss. When categorized, the subtotal aOR was 1.04 (95% CI 0.98–1.11, *P* = 0.209, *I*^2^ = 0.0%) in the loss of one tooth group, while in the significant tooth loss group, the subtotal aOR was 1.50 (95% CI 1.15–1.95, *P* = 0.003, *I*^2^ = 0.0%). Heterogeneity still existed when the studies were divided according to study design, as demonstrated in Figure 3c. The subgroup analysis of pooled unadjusted ORs reported similar results, as shown in Supplementary Figures S3-3, S3-4. A sensitivity analysis, using the leave-one-out method, indicated no single study significantly altered the results, as shown in Figure 3d.

## Discussion

Our meta-analysis, which incorporated fully adjusted pooled ORs, revealed no significant association between periodontitis or tooth loss and MCI in elderly patients, despite all the unadjusted ORs being statistically significant. The subgroup analyses also yielded statistically significant pooled ORs. When elderly patients with periodontitis were diagnosed using the CDC-AAP criteria, their risk of developing MCI was nearly 1.8 times higher than that of those without periodontitis; similarly, older adults with significant tooth loss were found to have a 1.5 times higher risk of progressing to MCI compared with those without. Thus, it remains meaningful for researchers to emphasize the importance of strengthening periodontal health management and providing high-quality oral health care services to older adults in preventing MCI, AD, and dementia, since approximately 70% of dementia cases are caused by AD, and 45% of cases are preventable. Dementia, one of the world's deadliest non-communicable diseases (59), has gained rising global awareness due to its increased prevalence, pathophysiological irreversibility, clinical incurable characteristics, reduced life quality and expectancy, and heavy global financial burden (60). As reported in 2025, the incidence rate of dementia in China is outpacing the global average (61).

Our results showed no correlation between periodontitis, tooth loss, and MCI, which is different from previous meta-analyses (4, 25–28, 30, 62, 63). This may be due to differences in study design, the population's exposure of interest, and the relationship between exposure and outcome. In our research, we focused on the early phase of cognitive impairment that progresses to AD or dementia. In order to clarify a single-direction correlation between periodontitis, tooth loss, and MCI, we included cohort, cross-sectional, and randomized controlled studies (the latter were not available), but no case-control studies. In addition, our study aimed to incorporate research with coexisting exposure to severe periodontitis and tooth loss to elucidate whether tooth loss among patients with periodontitis is a significant risk factor for MCI. Unfortunately, this analysis could not be undertaken due to inadequate available data; however, Qadir et al. identified an aOR of 1.98 for MCI in their systematic umbrella meta-analysis (63). Although we did not find the same high level of MCI risk as in the meta-analysis of Agarwal et al. (25), our findings supported the significantly adverse effect of both severe periodontitis and significant tooth loss on MCI. As previous studies reported (63–65), a strong correlation was found between severe periodontitis and the incidence of MCI in the elderly.

Our subgroup analysis revealed that the severity of periodontitis and tooth loss exerted a profound influence on MCI outcomes. Specifically, the most severe forms of periodontitis and tooth loss were strongly associated with the incidence of MCI. Several reasons may explain the adverse effects of severe periodontitis and significant tooth loss on MCI risk. Oral disease that has progressed from periodontitis to the final stage of tooth loss may increase MCI risk through the “nutrition pathway” (49). Periodontitis and tooth loss result in reduced chewing function, which leads to poorer digestive capacity and appetite. This decreased nutrition then ultimately damages brain functions such as cognition, memory, and even emotion. Dietary interventions for the deficiency of several nutrients, such as vitamin D and iron deficiencies, also seemed to be associated with a lower risk of cognitive dysfunction. Patients with tooth loss are less likely to eat a fiber-enriched diet due to impaired dentition and chewing difficulty; thus, the saturated fat- and meat-enriched, serum albumin-lacking diet pattern is pro-inflammatory, and finally leads to a detrimental impact on their cognitive function (66). Moreover, a few interventional studies have proved causality between vitamin D and iron deficiencies and the risk of dementia (67, 68). Elevated plasma Aβ_1–40_ or Tau may be two blood-based key biomarkers in patients with periodontitis for the progression from cognitive impairment to AD (69). Gao et al. found robust bidirectional associations between periodontitis, tooth loss, dental caries, and poor cognitive function in an aging population (49, 70), while Holmer et al. found no associations (71). Tooth loss and periodontitis are mutual promoters in oral disease progression. Specific neuroinflammation mechanisms may also be linked to periodontitis (72).

### Strengths and limitations

One of the strengths of our study is that we collected data from patients with both tooth loss and periodontitis at the same time, since not all tooth loss is caused by periodontitis. Instead of self-reported tooth loss or periodontitis without confirmation, all tooth loss and periodontitis diagnoses were made using objective methods by at least two qualified dental specialists. Second, we performed subgroup and sensitivity analyses to evaluate the effect of the severity of tooth loss and periodontitis. It was found that the same diagnostic or evaluating standards had a notable impact on decreasing heterogeneity among different studies. Although the loss of one tooth had a mild effect on developing mild cognitive impairment, the subgroup analysis showed that the loss of five or more teeth profoundly increases the possibility of MCI. Third, we tried to omit each study stepwise in the sensitivity analysis to assess whether the heterogeneity was a result of any of the included studies. The unchanged results indicated a robust association and that the heterogeneity did not originate from any single study.

This study had several limitations. The majority of the included studies were cross-sectional or cohort studies, making it difficult to establish a causal relationship between oral disease and MCI. Additionally, due to insufficient data from fewer than three original studies, we were unable to assess the dose–response relationship between periodontitis or tooth loss and risk of MCI or dementia due to a lack of data from more than two original studies. A meta-regression analysis to evaluate the influence of moderators (e.g., study design) was not feasible due to the small number of included studies (*n* = 8). Meta-regression typically requires a larger sample of at least 10 studies to ensure a robust assessment of moderator effects, and we were limited by the scarcity of eligible research on this specific topic. Since both periodontitis and MCI were diagnosed according to different criteria, high heterogeneity between the included studies was observed. Additionally, publication bias was another source of heterogeneity. Due to the limited number of included studies, we could n't conduct an assessment of publication bias (50). Finally, given the observational nature of the studies with numerous confounding factors, we chose aOR for the primary analysis. However, even with multiple adjustments, confounding effects cannot be completely avoided.

## Conclusion

Our meta-analysis found no significant overall association between periodontitis, tooth loss, and increased MCI incidence. However, the MCI incidence was significantly higher in patients with severe periodontitis and severe tooth loss. Therefore, maintaining intact tooth structure without the progression of new caries, along with minimal or no periodontal inflammation, could be valuable for preventing cognitive decline.

## Data Availability

The raw data supporting the conclusions of this article will be made available by the authors, without undue reservation.

## Supplementary material

Supplementary Appendix 1 Search strategy.

Supplementary Appendix 2 Excluded studies and reasons.

Supplementary Appendix 3 The Newcastle–Ottawa scale used for included studies’ quality evaluation.

Supplementary Appendix 4 Meta-analysis of pooled unadjusted ORs of included studies.
