# Supplementary material for: An updated systematic review and meta-analysis of tooth loss in patients with periodontitis and the risk of mild cognitive impairment
Source: Front Oral Health. 2026 Mar 18;7:1710871. doi: 10.3389/froh.2026.1710871 (PMC13039103; doi:10.3389/froh.2026.1710871)

**FIGURE 1.** Flowchart of the included studies in this systematic review.


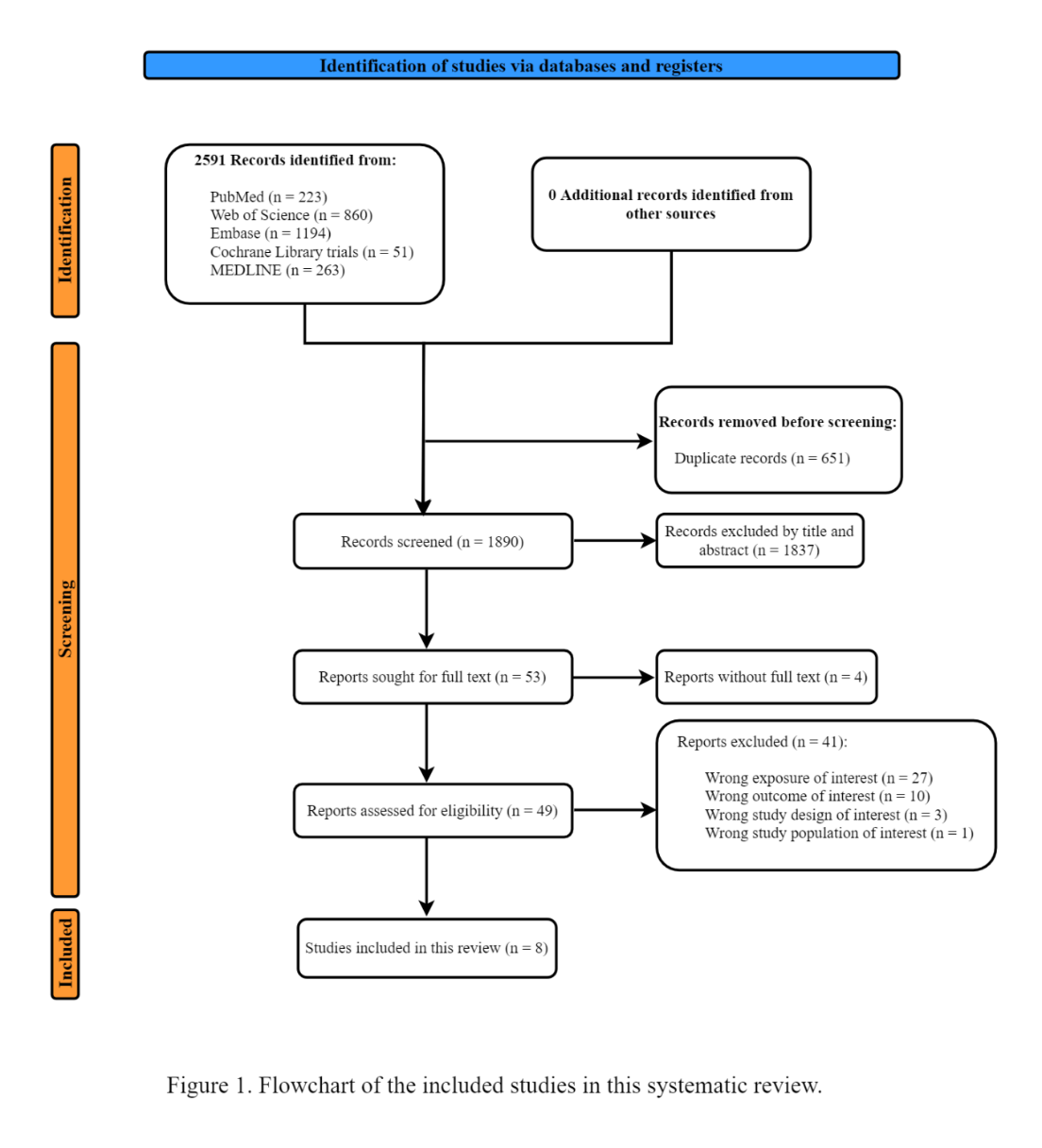


**FIGURE 2.** Meta analysis of association between periodontitis and MCI. (**FIGURE 2-1.** Forest plot of association between periodontitis and MCI. **FIGURE 2-2.** Subgroup analysis for association between periodontitis and MCI. **FIGURE 2-3.** Subgroup analysis for association between periodontitis and MCI. **FIGURE 2-4.** Sensitivity analysis of influence of periodontitis on MCI.)


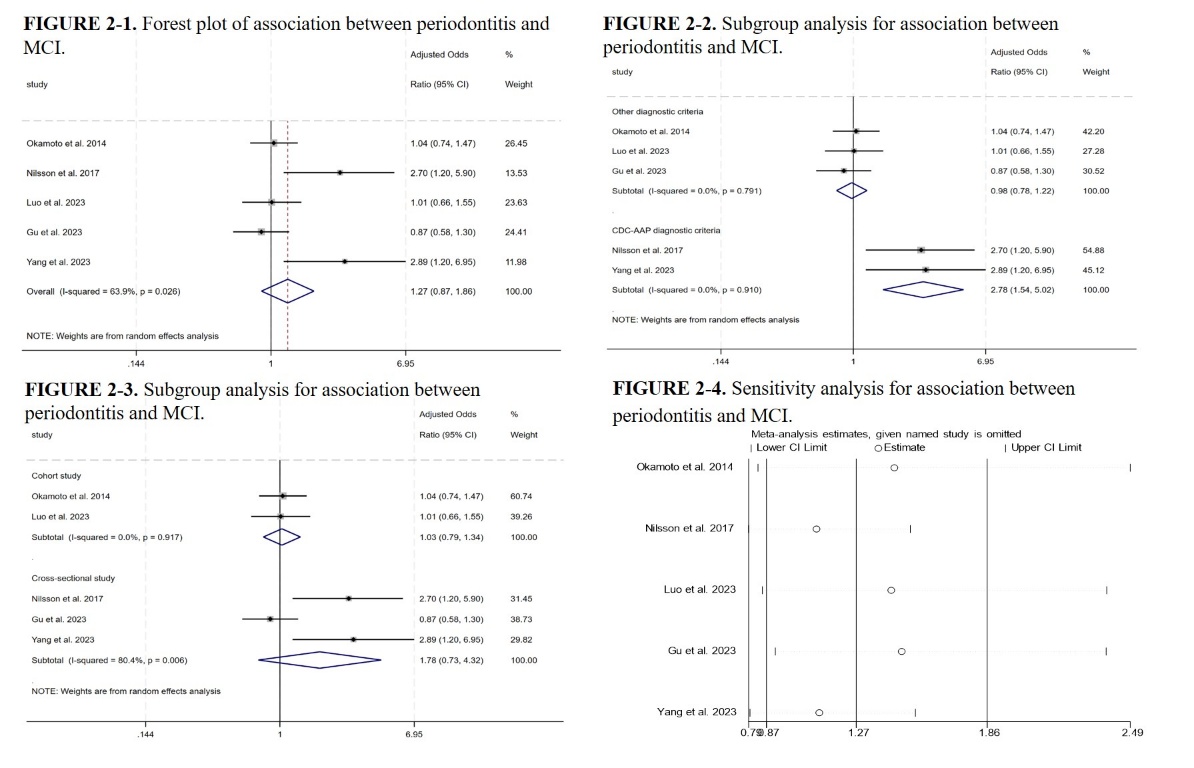


**FIGURE 3.** Meta analysis of association between tooth loss and MCI. (**FIGURE 3-1.** Forest plot of association between tooth loss and MCI. **FIGURE 3-2.** Subgroup analysis for association between tooth loss and MCI. **FIGURE 3-3.** Subgroup analysis for association between tooth loss and MCI. **FIGURE 3-4.** Sensitivity analysis of influence of tooth loss on MCI.)


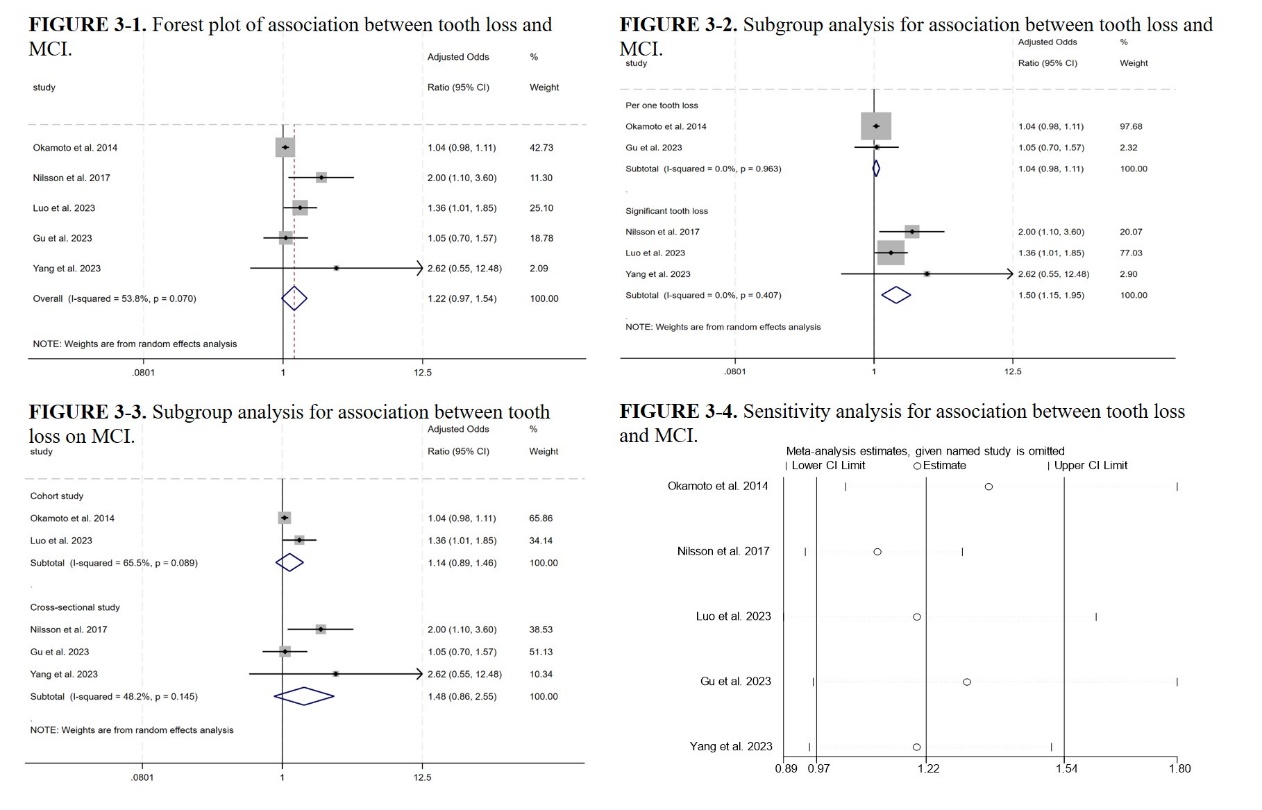

Supplement: Supplementary Appendix 1 — Search strategy. [file Supplementaryfile1.docx]
