# Supplementary material for: An updated systematic review and meta-analysis of tooth loss in patients with periodontitis and the risk of mild cognitive impairment
Source: Front Oral Health. 2026 Mar 18;7:1710871. doi: 10.3389/froh.2026.1710871 (PMC13039103; doi:10.3389/froh.2026.1710871)

Supplemental Appendix

**An updated systematic review and meta-analysis for tooth loss in periodontitis patients and risk of mild cognitive dysfunction**

Table of contents

Supplemental Appendix 1: Search strategy

Supplemental Appendix 2: Excluded studies and reasons

Supplemental Appendix 3: The Newcastle-Ottawa scale used for included studies’ quality evaluation.

Supplemental Appendix 4: Meta analysis of pooled unadjusted ORs of included studies.

**Supplemental Appendix 1: Search strategy.**

**Table S1** Search strategy of PubMed

| # | Searches | Number |
| --- | --- | --- |
| 1 | (((((tooth loss[MeSH Terms]) OR (tooth[Title/Abstract])) OR (missing tooth[Title/Abstract])) OR (no tooth[Title/Abstract])) OR (edentulism dentition[Title/Abstract])) OR (loss of teeth[Title/Abstract]) | 120367 |
| 2 | ((((((periodontitis[MeSH Terms]) OR (chronic periodontitis[MeSH Terms])) OR (periodontal disease[Title/Abstract])) OR (chronic periodontal diseases[Title/Abstract])) OR (chronic periodontitis[Title/Abstract])) OR (periodontitis[Title/Abstract])) OR (dental caries[Title/Abstract]) | 91453 |
| 3 | ((((((((cognitive dysfunction[MeSH Terms]) OR (mild cognitive impairment[Title/Abstract])) OR (cognitive decline[Title/Abstract])) OR (cognitive deterioration[Title/Abstract])) OR (mental deterioration[Title/Abstract])) OR (mild neurocognitive disorder[Title/Abstract])) OR (mild memory loss[Title/Abstract])) OR (mild memory impairment[Title/Abstract])) OR (mild amnesia[Title/Abstract]) | 90562 |
| 4 | #1 OR #2 | 196474 |
| 5 | #3 AND #4 | 296 |
| 6 | ((((meta analysis[MeSH Terms]) OR (meta analysis as topic[MeSH Terms])) OR (meta analysis[Publication Type])) OR (review[Publication Type])) OR (meta-analysis[Title/Abstract]) | 3749548 |
| 7 | #5 NOT #6 | 223 |

**Table S2** Search strategy of Web of Science

| # | Searches | Number |
| --- | --- | --- |
| 1 | ((((((((((TS=(tooth loss)) OR TS=(missing tooth)) OR TS=(no tooth)) OR TS=(edentulism dentition)) OR TS=(loss of teeth)) OR TS=(periodontitis)) OR TS=(chronic periodontitis)) OR TS=(periodontal disease*)) OR TS=(chronic periodontal disease*)) OR TS=(chronic periodontitis)) OR TS=(dental caries) | 369754 |
| 2 | ((((((((TS=(cognitive dysfunction)) OR TS=(mild cognitive impairment)) OR TS=(cognitive decline)) OR TS=(cognitive deterioration)) OR TS=(mental deterioration)) OR TS=(mild neurocognitive disorder)) OR TS=(mild memory loss)) OR TS=(mild memory impairment)) OR TS=(mild memory impairment) | 311462 |
| 3 | #1 AND #2 | 1051 |
| 4 | ((TS=(meta*analysis)) OR TS=(meta analysis as topic)) OR TS=(review) | 5926624 |
| 5 | #3 NOT #4 | 860 |

**TableS3** Search strategy of Embase.

| # | Searches | Number |
| --- | --- | --- |
| 1 | 'periodontal disease'/exp OR 'periodontal disease' OR 'edentulism'/exp OR 'edentulism' | 150962 |
| 2 | 'tooth loss':ti,ab,kw OR 'missing tooth':ti,ab,kw OR 'no tooth':ti,ab,kw OR 'edentulism dentition':ti,ab,kw OR 'loss of teeth':ti,ab,kw | 9025 |
| 3 | 'periodontal disease'/exp OR 'periodontal disease' OR (periodontal AND ('disease'/exp OR disease)) OR 'chronic periodontal diseases':ti,ab,kw OR 'chronic periodontitis':ti,ab,kw OR periodontitis:ti,ab,kw OR 'dental caries':ti,ab,kw | 182620 |
| 4 | #1 OR #2 OR #3 | 194088 |
| 5 | 'cognitive defect'/exp | 692632 |
| 6 | 'cognitive dysfunction'/exp OR 'cognitive dysfunction' OR (cognitive AND dysfunction) OR 'mild cognitive impairment':ti,ab,kw OR 'cognitive decline':ti,ab,kw OR 'cognitive deterioration':ti,ab,kw OR 'mental deterioration':ti,ab,kw OR 'mild neurocognitive disorder':ti,ab,kw OR 'mild memory loss':ti,ab,kw OR 'mild memory impairment':ti,ab,kw OR 'mild amnesia':ti,ab,kw OR 'cognition disorder':ti,ab,kw OR 'cognitive complaints':ti,ab,kw OR 'cognitive defects':ti,ab,kw OR 'cognitive problems':ti,ab,kw | 740418 |
| 7 | #5 OR #6 | 7740418 |
| 8 | #4 AND #7 | 1891 |
| 9 | 'meta analysis'/exp OR 'meta analysis' OR (meta AND ('analysis'/exp OR analysis)) OR 'meta analysis':it OR review:it | 3746336 |
| 10 | #8 NOT #9 | 1194 |

**TableS4** Search strategy of Cochranelibrary trials.

| # | Searches | Number |
| --- | --- | --- |
| 1 | MeSH descriptor:[Tooth Loss] explode all trees | 215 |
| 2 | (tooth loss):ti,ab,kw OR (missing tooth):ti,ab,kw OR (no tooth):ti,ab,kw OR(edentulism dentition):ti,ab,kw OR (loss of teeth):ti,ab,kw | 19130 |
| 3 | #1 OR #2 | 19130 |
| 4 | MeSH descriptor:[Periapical Periodontitis] explode all trees | 436 |
| 5 | MeSH descriptor:[Chronic Periodontitis] explode all trees | 1068 |
| 6 | (dental caries):ti,ab,kw OR (periodontitis):ti,ab,kw OR (chronic periodontitis):ti,ab,kw OR(chronic periodontal diseases):ti,ab,kw OR (periodontal disease):ti,ab,kw | 17679 |
| 7 | #4 or #5 or #6 | 17713 |
| 8 | #3 or #7 | 31166 |
| 9 | MeSH descriptor:[Cognition Disorders] explode all trees | 8702 |
| 10 | (mild cognitive impairment):ti,ab,kw OR (cognitive decline):ti,ab,kw OR (cognitive deterioration):ti,ab,kw OR(mental deterioration):ti,ab,kw OR (mild neurocognitive disorder):ti,ab,kw | 13444 |
| 11 | (mild memory loss):ti,ab,kw OR (mild memory impairment):ti,ab,kw OR (mild amnesia):ti,ab,kw OR(cognitive defects):ti,ab,kw OR (cognitive problems):ti,ab,kw | 10715 |
| 12 | #9 or #10 or #11 | 26080 |
| 13 | #8 and #12 | 56 |
| 14 | Filter 1: Trials | 51 |

**TableS5** Search strategy of MEDLINE.

| # | Searches | Number |
| --- | --- | --- |
| 1 | tooth loss.sh. | 4802 |
| 2 | (tooth loss or missing tooth or no tooth or edentulism dentition or loss of teeth).mp. [mp=title, book title, abstract, original title, name of substance word, subject heading word, floating sub-heading word, keyword heading word, organism supplementary concept word, protocol supplementary concept word, rare disease supplementary concept word, unique identifier, synonyms, population supplementary concept word, anatomy supplementary concept word] | 10655 |
| 3 | #1 OR #2 | 10655 |
| 4 | periodontitis.sh. | 23088 |
| 5 | chronic periodontitis.sh. | 3912 |
| 6 | (periodontal disease or chronic periodontal diseases or chronic periodontitis or periodontitis or dental caries).mp. [mp=title, book title, abstract, original title, name of substance word, subject heading word, floating sub-heading word, keyword heading word, organism supplementary concept word, protocol supplementary concept word, rare disease supplementary concept word, unique identifier, synonyms, population supplementary concept word, anatomy supplementary concept word] | 119118 |
| 7 | #4 OR #5 OR #6 | 119118 |
| 8 | cognitive dysfunction.sh. | 46083 |
| 9 | (mild cognitive impairment or cognitive decline or cognitive deterioration or mental deterioration or mild neurocognitive disorder or mild memory loss or mild memory impairment or mild amnesia or cognition disorder or cognitive complaints or cognitive defects or cognitive problems).mp. [mp=title, book title, abstract, original title, name of substance word, subject heading word, floating sub-heading word, keyword heading word, organism supplementary concept word, protocol supplementary concept word, rare disease supplementary concept word, unique identifier, synonyms, population supplementary concept word, anatomy supplementary concept word] | 71940 |
| 10 | #8 OR #9 | 94345 |
| 11 | #3 OR #7 | 125234 |
| 12 | #10 AND #11 | 280 |
| 13 | meta analysis.pt. or meta analysis.ti. or meta analysis.ab. or meta$analysis.kw. or review.kw. | 344530 |
| 14 | #12 NOT #13 | 263 |

**Supplemental Appendix 2** **Excluded studies and reasons.**

| **Number** | **Study** | **Reason** |
| --- | --- | --- |
| 1 | Chen J-T, Tsai S, Chen M-H, Pitiphat W, Matangkasombut O, Chiou J-M, Han M-L, Chen J-H, Chen Y-C: Association between oral health and cognitive impairment in older adults: Insights from a Six-year prospective cohort study. Journal of Dentistry 2024, 147.[1] | The study mainly described dental caries as the primary reason for MCI. The exposure was different. |
| 2 | Chen HM, Li KY, Li TL, Wong GHY, Kwong YL, Ng RC-L, Burrow MF, McGrath C, Chen H: Association of dietary inflammation with tooth loss and cognitive decline in older adults from cross-sectional data: The moderated role of albumin. Journal of Dentistry 2024, 144.[2] | The outcome was score for cognitive impairment, without diagnosis of MCI. The outcome was different. |
| 3 | Chen HM, Li KY, Li TL, Kwong E, Wong GHY, McGrath C, Chen H: The association between tooth loss and cognitive decline in the aged population: The mediating role of HDL-cholesterol. Journal of Dentistry 2023, 135.[3] | In this study, the exposure was only periodontitis without tooth loss. The exposure was different. |
| 4 | Jones JA, Moss K, Finlayson TL, Preisser JS, Weintraub JA: Edentulism Predicts Cognitive Decline in the US Health and Retirement Cohort Study. Journal of Dental Research 2023, 102(8):863-870.[4] | Only periodontitis was exposure in the study, tooth loss was not found as exposure. The exposure was different. |
| 5 | Asher S, Suominen AL, Stephen R, Ngandu T, Koskinen S, Solomon A: Association of tooth count with cognitive decline and dementia in the Finnish adult population. Journal of Clinical Periodontology 2023, 50(9):1154-1166.[5] | In this study, the exposure was only periodontitis without tooth loss. The exposure was different. |
| 6 | Carballo Á, López‐Dequidt I, Custodia A, Botelho J, Aramburu‐Núñez M, Machado V, Pías‐Peleteiro JM, Ouro A, Romaus‐Sanjurjo D, Vázquez‐Vázquez L et al: Association of periodontitis with cognitive decline and its progression: Contribution of blood‐based biomarkers of Alzheimer's disease to this relationship. Journal of Clinical Periodontology 2023, 50(11):1444-1454.[6] | only periodontitis was exposure in the study, tooth loss was not found as exposure. The exposure was different. |
| 7 | Marruganti C, Baima G, Aimetti M, Grandini S, Sanz M, Romandini M: Periodontitis and low cognitive performance: A population‐based study. Journal of Clinical Periodontology 2023, 50(4):418-429.[7] | In this study, the exposure was only periodontitis without tooth loss. The exposure was different. |
| 8 | Saji N, Ishihara Y, Murotani K, Uchiyama A, Takeda A, Sakurai T, Matsushita K: Cross-Sectional Analysis of Periodontal Disease and Cognitive Impairment Conducted in a Memory Clinic: The Pearl Study. Journal of Alzheimer’s Disease 2023, 96(1):369-380.[8] | The outcome was dementia, and mild cognitive impairment was categorized into non-dementia without exact sample size. The outcome was different. |
| 9 | He F, Luo H, Yin L, Roosaar A, Axéll T, Zhao H, Ye W: Poor Oral Health as a Risk Factor for Dementia in a Swedish Population: A Cohort Study with 40 Years of Follow-Up. Journal of Alzheimer's Disease 2023, 92(1):171-181.[9] | The outcome was dementia, not mild cognitive impairment. The outcome was different. |
| 10 | Yoo JE, Huh Y, Park S-H, Han K, Park HS, Cho KH, Ahn J-S, Jun SH, Nam GE: Association between Dental Diseases and Oral Hygiene Care and the Risk of Dementia: A Retrospective Cohort Study. Journal of the American Medical Directors Association 2023, 24(12):1924-1930.e1923.[10] | The outcome was dementia and Alzheimer’s disease, not mild cognitive impairment. The outcome was different. |
| 11 | Kulkarni MS, Miller BC, Mahani M, Mhaskar R, Tsalatsanis A, Jain S, Yadav H: Poor Oral Health Linked with Higher Risk of Alzheimer’s Disease. Brain Sciences 2023, 13(11).[11] | The outcome was Alzheimer’s disease, not mild cognitive impairment. The outcome was different. |
| 12 | Xiang Q, Zheng Z, Yaolin P, Bei W: Denture use and a slower rate of cognitive decline among older adults with partial tooth loss in China: A 10-year prospective cohort study. Aging Med (Milton) 2025, 7(6).[12] | In this study, the exposure was only tooth loss without periodontitis. The exposure was different. |
| 13 | Chou Y-C, Weng S-H, Cheng F-S, Hu H-Y, Lipsitz LA: Denture Use Mitigates the Cognitive Impact of Tooth Loss in Older Adults. The Journals of Gerontology, Series A: Biological Sciences and Medical Sciences 2025, 80(1).[13] | In this study, the exposure was only tooth loss without periodontitis. The exposure was different. |
| 14 | Yang Q, Zhang J-h, Mao J, Zeng T-y, Tian S-w: Functional teeth and cognitive function among the Chinese elderly: The chain mediating effect of depressive symptoms and social participation. Geriatric Nursing 2024, 58:111-118.[14] | In this study, the exposure was only tooth loss without periodontitis. The exposure was different. |
| 15 | Rajendran V, Uppoor A, Nayak SU, Rao SB, Dasson Bajaj P: Unraveling the cognitive implications among individuals with co-occurring chronic periodontitis and type 2 diabetes mellitus: A cross-sectional study. Journal of Oral Biosciences 2024, 66(3):605-611.[15] | the exposure was periodontitis and diabetes, but without tooth loss. The exposure was different. |
| 16 | Budală DG, Balcoș C, Armencia A, Virvescu DI, Lupu CI, Baciu ER, Vasluianu RI, Tatarciuc M, Luchian I: Does the Loss of Teeth Have an Impact on Geriatric Patients’ Cognitive Status? Journal of Clinical Medicine 2023, 12(6).[16] | Periodontitis or tooth loss was not considered as primary exposure of the study. The exposure was different. |
| 17 | Li Y, Huang C-L, Lu X-Z, Tang Z-Q, Wang Y-Y, Sun Y, Chen X: Longitudinal association of edentulism with cognitive impairment, sarcopenia and all-cause mortality among older Chinese adults. BMC Oral Health 2023, 23(1).[17] | The exposure in this study was edentulism without periodontitis. The exposure was different. |
| 18 | Nazar G, Díaz-Toro F, Roa P, Petermann-Rocha F, Troncoso-Pantoja C, Leiva-Ordóñez AM, Cigarroa I, Celis-Morales C: Asociación entre salud oral y deterioro cognitivo en personas mayores chilenas. Gaceta Sanitaria 2023, 37(0).[18] | In this study, the exposure was only tooth loss without periodontitis. The exposure was different. |
| 19 | Wu B, Luo H, Tan C, Qi X, Sloan FA, Kamer AR, Schwartz MD, Martinez M, Plassman BL: Diabetes, Edentulism, and Cognitive Decline: A 12-Year Prospective Analysis. Journal of Dental Research 2023, 102(8):879-886.[19] | In this study, the exposure was edentulism with diabetes. The exposure was different. |
| 20 | Brahmbhatt Y, Alqaderi H, Chinipardaz Z: Association Between Severe Periodontitis and Cognitive Decline in Older Adults. Life 2024, 14(12).[20] | In this study, the exposure was only periodontitis without tooth loss. The exposure was different. |
| 21 | Deng Z, Li J, Zhang Y, Zhang Y: No genetic causal associations between periodontitis and brain atrophy or cognitive impairment: evidence from a comprehensive bidirectional Mendelian randomization study. BMC Oral Health 2024, 24(1).[21] | In this study, the exposure was only periodontitis without tooth loss. The exposure was different. |
| 22 | Igase M, Igase K, Hino S, Uchida D, Okada Y, Ochi M, Tabara Y, Ohyagi Y: Association of Periodontitis with Mild Cognitive Impairment in Older Adults. The Journal of Aging Research & Lifestyle 2024, 13(0):108-112.[22] | In this study, the exposure was only periodontitis without tooth loss. The exposure was different. |
| 23 | Kiuchi S, Matsuyama Y, Takeuchi K, Kusama T, Cooray U, Osaka K, Aida J: Number of Teeth and Dementia-free Life Expectancy: A 10-Year Follow-Up Study from the Japan Gerontological Evaluation Study. Journal of the American Medical Directors Association 2024, 25(11).[23] | In this study, the exposure was only tooth loss without periodontitis. The exposure was different. |
| 24 | Lee KY, Chan CCK, Yip C, Li JTW, Hau CF, Poon SSY, Chen HM, Li KY, Burrow MF, Wong GHY et al: Association between tooth loss‐related speech and psychosocial impairment with cognitive function: A pilot study in Hong Kong's older population. Journal of Oral Rehabilitation 2024, 51(8):1475-1485.[24] | In this study, the exposure was tooth loss‐related speech and psychosocial impairment but not tooth loss and periodontitis. The exposure was different. |
| 25 | Thu Ya M, Hasegawa Y, Sta. Maria MT, Hattori H, Kusunoki H, Nagai K, Tamaki K, Hori K, Kishimoto H, Shinmura K: Predicting cognitive function changes from oral health status: a longitudinal cohort study. Scientific Reports 2024, 14(1).[25] | In this study, the exposure was only tooth loss without periodontitis. The exposure was different. |
| 26 | Shang D, Williams C, Vu G, Joshi A: Teeth, Health, and Mind: Understanding the Interplay of Social Determinants and Cognitive Decline in Older Adults. Journal of Applied Gerontology 2024(0).[26] | Tooth loss and periodontitis were not the primary exposure of this study. The exposure was different. |
| 27 | Khalaila R, Cohn-Schwartz E, Shiovitz-Ezra S, Lawlor B: A prospective association between social isolation and cognitive performance among older adults in Europe: the role of loneliness and poor oral health. Aging & Mental Health 2024, 28(8):1162-1168.[27] | Social isolation was the exposure of this study. The exposure was different. |
| 28 | Kusama T, Takeuchi K, Kiuchi S, Aida J, Osaka K: Poor oral health and dementia risk under time‐varying confounding: A cohort study based on marginal structural models. Journal of the American Geriatrics Society 2023, 72(3):729-741.[28] | The outcome was dementia, mild cognitive impairment was a covariate in this study. The outcome was different. |
| 29 | Matsuyama Y: Time‐varying exposure analysis of the relationship between sustained natural dentition and cognitive decline. Journal of Clinical Periodontology 2023, 50(6):727-735.[29] | In this study, the exposure was only tooth loss without periodontitis. The exposure was different. |
| 30 | Zhao D, Luo J, Li J, Gao T, Fu P, Wang Y, Zhou C: Tooth loss, body mass index and cognitive function among middle-aged and older adults in China: Does gender matter? Journal of Affective Disorders 2023, 333(0):517-523.[30] | The exposure was BMI but not tooth loss or periodontitis. The exposure was different. |
| 31 | Qi X, Zhu Z, Wang K, Zheng Y, Li A, Wu B: Association of Gum Treatment with Cognitive Decline and Dementia Risk among Older Adults with Periodontal Symptoms: A 12-Year Prospective Cohort Study. Neuroepidemiology 2024(0):1-10.[31] | The exposure was gum treatment with cognitive decline. The exposure was different. |
| 32 | Chen H-L, Wu D-R, Chen J-J, Lin W-S, Chen IC, Liu J-F, Lien S, Lin C-H: Association between periodontitis treatment and dementia in Taiwanese adults. BMC Oral Health 2023, 23(1).[32] | Case-control study. The study design was different. |
| 33 | Zhang R-Q, Ou Y-N, Huang S-Y, Li Y-Z, Huang Y-Y, Zhang Y-R, Chen S-D, Dong Q, Feng J-F, Cheng W et al: Poor Oral Health and Risk of Incident Dementia: A Prospective Cohort Study of 425,183 Participants. Journal of Alzheimer's Disease 2023, 93(3):977-990.[33] | The outcome was incident dementia. The outcome was different. |
| 34 | Zhang H, Sun L, Zhang L, Li J, Liu Y, Chen Z, Wang S, Gao C, Sun X: The role of periodontitis in the link between alpha-tocopherol intake and cognitive performance: A mediation analysis in older adults. Frontiers in Aging Neuroscience 2023, 15(0).[34] | Periodontitis was mediation factor but not exposure in this study, the exposure was alpha-tocopherol. The exposure was different. |
| 35 | Shiraki H, Kakuta S, Park J-W, Aosa T, Ansai T: Influence of Age on Associations of Occlusal Status and Number of Present Teeth with Dementia in Community-Dwelling Older People in Japan: Cross-Sectional Study. International Journal of Environmental Research and Public Health 2023, 20(9).[35] | The outcome was dementia, the exposure in this study was occlusal status. The exposure and outcome were different. |
| 36 | Karaduran K, Aydogdu A, Gelisin O, Gunpinar S: Investigating the potential clinical impact of periodontitis on the progression of Alzheimer’s disease: a prospective cohort study. Clinical Oral Investigations 2023, 28(1).[36] | Case-control study. The study design was different. |
| 37 | Cerajewska TL, Davies M, Allen-Birt SJ, Swirski M, Coulthard EJ, West NX: A feasibility study to recruit, retain and treat periodontitis in volunteers with mild dementia, whilst monitoring their cognition. Journal of Dentistry 2024, 150(0).[37] | The population in this study were people with both mild dementia and periodontal disease. The population were different. |
| 38 | Hu C, Li H, Huang L, Wang R, Wang Z, Ma R, Chang B, Li S, Li H, Li G: Periodontal disease and risk of Alzheimer's disease: A two‐sample Mendelian randomization. Brain and Behavior 2024, 14(4).[38] | The outcome was Alzheimer’s disease, but not mild cognitive impairment. The outcome was different. |
| 39 | Ji X, Wu Y, Gu Z, Zhong Z, Wang K, Ye S, Wan Y, Qiu P: Trajectories of cognitive function and frailty in older adults in China: a longitudinal study. Frontiers in Aging Neuroscience 2024, 16(0).[39] | The outcome was frailty but not mild cognitive impairment. The outcome was different. |
| 40 | Kim KS, Ahn S, Han JW, Cho SH, Lee JT, Lee HJ, Kim KW: Oral health and risk of cognitive disorders in older adults: A biannual longitudinal follow‐up cohort. Journal of Oral Rehabilitation 2023, 50(9):792-801.[40] | The exposure in this study were masticatory performance and removable partial denture use. The exposure was different. |

**Supplemental Appendix 3** Evaluation of the Newcastle-Ottawa scale for quality of the included 8 studies.

| **Research** | **A** | **B** | **C** | **D** | **E** | **F** | **G** | **H** | **I** | **Total (maximum possible score=9)** | **Quality grade** |
| --- | --- | --- | --- | --- | --- | --- | --- | --- | --- | --- | --- |
| Kaye et al. 2010 | * | * | * | - | * | * | * | * | * | 8 | High |
| Okamoto et al. 2014 | - | - | * | * | * | * | - | * | - | 5 | Low |
| Nilsson et al. 2017 | * | * | - | - | - | * | * | * | - | 5 | Moderate |
| Luo et al. 2023 | * | * | * | * | * | * | * | * | - | 8 | High |
| Gu et al. 2023 | * | * | * | * | - | * | * | * | * | 8 | High |
| Yang et al. 2023 | * | * | * | - | - | * | * | - | * | 6 | Moderate |
| Gao et al. 2024 | * | * | * | * | - | * | * | - | * | 7 | High |
| Ye et al. 2024 | * | * | * | * | - | * | * | - | * | 7 | High |

A, represent “Representativeness of the exposed cohort”; B, represent “Selection of the non-exposed cohort”; C, represent “Ascertainment of exposure (periodontitis and/or tooth loss)”; D, represent “Demonstration that the outcome (dementia/cognitive impairment) was not present at the start of the study”; E, represent “Comparability of cohorts according to the design and analysis”; F, represent “The study controls for additional factors (age, diet, smoking, education, socioeconomic factors, etc.)”; G, represent “Assessment of mild-cognitive impairment using validated assessment tools such as MMSE or the six-item screener test”; H, represent “Follow-up was long enough for the outcomes to occur”; I, represent “adequacy of follow-up of cohorts”. A to D were clarified as “the selection domain”; E to F were clarified as “the comparability domain”; G to I were clarified as “the outcome/exposure domain”.

Legenda: (*) stands for present.

**Supplemental Appendix 4** Meta analysis of pooled unadjusted ORs of included studies.


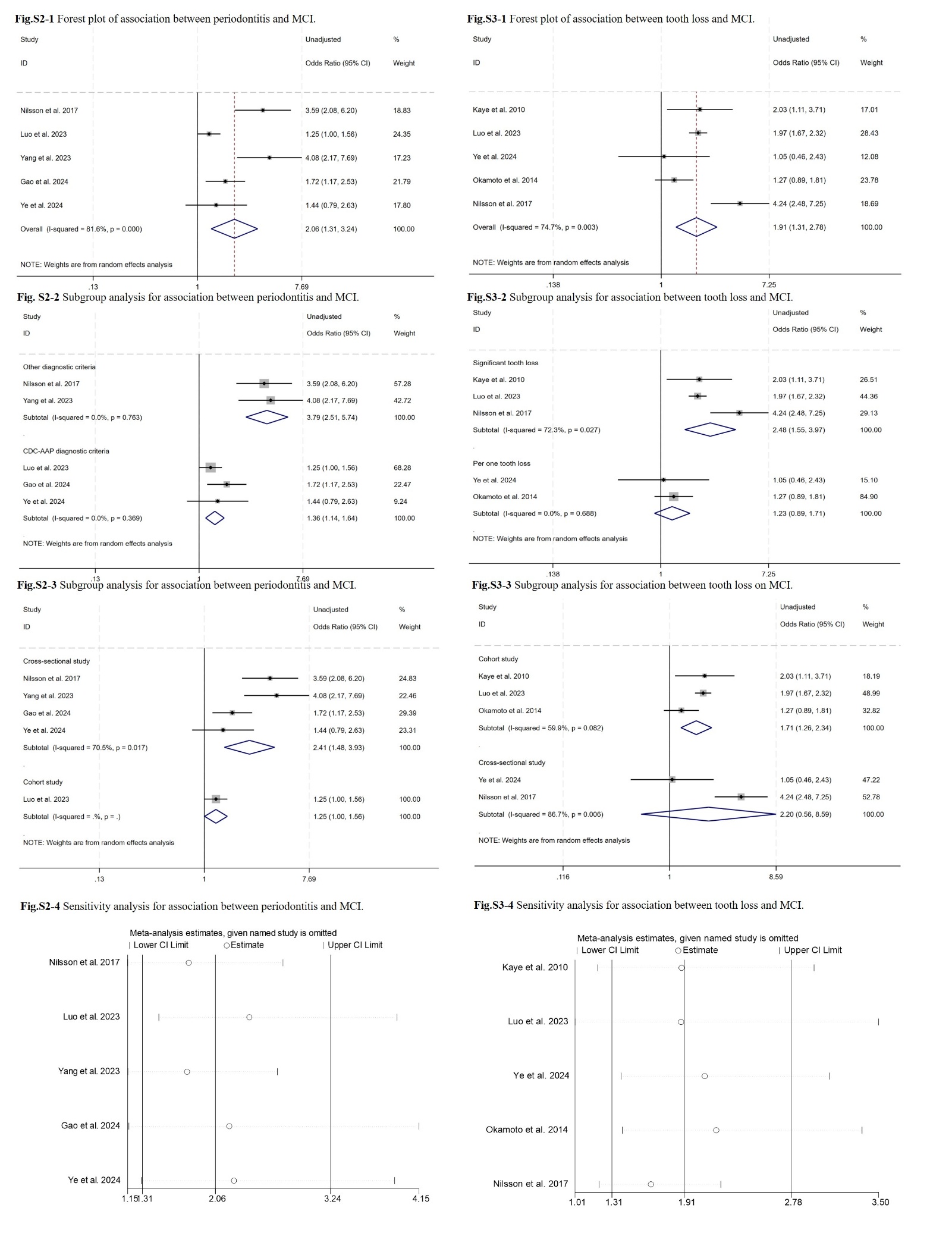

Supplement: Supplementary Appendix 2 — Excluded studies and reasons. [file Supplementaryfile2.docx]
